# Exploring the relationship between tychoparthenogenesis and inbreeding depression in the Desert Locust, *Schistocerca gregaria*

**DOI:** 10.1002/ece3.3103

**Published:** 2017-06-28

**Authors:** Chelsea J. Little, Marie‐Pierre Chapuis, Laurence Blondin, Elodie Chapuis, Hélène Jourdan‐Pineau

**Affiliations:** ^1^ Department of Aquatic Ecology Eawag: Swiss Federal Institute of Aquatic Science and Technology Dübendorf Switzerland; ^2^ UMR CBGP CIRAD Montpellier France; ^3^ UPR B‐AMR CIRAD Montpellier France; ^4^ IRD, Cirad Univ Montpellier, IPME Montpellier France; ^5^ UMR PVBMT CIRAD Saint‐Pierre La Réunion France

**Keywords:** desert locust, inbreeding depression, parthenogenesis, *Schistocerca gregaria*

## Abstract

Tychoparthenogenesis, a form of asexual reproduction in which a small proportion of unfertilized eggs can hatch spontaneously, could be an intermediate evolutionary link in the transition from sexual to parthenogenetic reproduction. The lower fitness of tychoparthenogenetic offspring could be due to either developmental constraints or to inbreeding depression in more homozygous individuals. We tested the hypothesis that in populations where inbreeding depression has been purged, tychoparthenogenesis may be less costly. To assess this hypothesis, we compared the impact of inbreeding and parthenogenetic treatments on eight life‐history traits (five measuring inbreeding depression and three measuring inbreeding avoidance) in four laboratory populations of the desert locust, *Schistocerca gregaria*, with contrasted demographic histories. Overall, we found no clear relationship between the population history (illustrated by the levels of genetic diversity or inbreeding) and inbreeding depression, or between inbreeding depression and parthenogenetic capacity. First, there was a general lack of inbreeding depression in every population, except in two populations for two traits. This pattern could not be explained by the purging of inbreeding load in the studied populations. Second, we observed large differences between populations in their capacity to reproduce through tychoparthenogenesis. Only the oldest laboratory population successfully produced parthenogenetic offspring. However, the level of inbreeding depression did not explain the differences in parthenogenetic success between all studied populations. Differences in development constraints may arise driven by random and selective processes between populations.

## 1 INTRODUCTION

Parthenogenesis has evolved independently in many animal species either as an obligatory or facultative form of reproduction. Among types of parthenogenesis, arrhenotoky (which produces haploid males) is always associated with sexual reproduction (De Meeûs, Prugnolle, & Agnew, 2007). On the contrary, thelytoky (which produces only females) as well as deuterotoky (producing females and nonreproductive males) allow for evolution toward obligate parthenogenesis in which sexual reproduction is suppressed (De Meeûs et al., 2007; Lenormand, Roze, Cheptou, & Maurice, 2016). On a cytological basis, two main types of thelytokous parthenogenesis can be distinguished. In apomictic parthenogenesis, meiosis is replaced by mitosis and the offspring are true clones of the mother. Alternatively, in automictic parthenogenesis, the first stages of meiosis occur but are followed by a fusion between two nuclei originating from the same individual. Different mechanisms maintain the ploidy level across generations of automictic parthenogenetic individuals: fusion of meiotic products, gamete duplication, or fusion of sister cells in the zygote. Each mode of thelytokous parthenogenesis has a different impact on inbreeding (Pearcy, Hardy, & Aron, 2006). Indeed, whereas automixis with fusion of meiotic products generates offspring that have a higher level of homozygosity than their mother, complete homozygosity is reached in the case of gamete duplication or fusion of sister cells. Automictic parthenogenesis could occur via tychoparthenogenesis, which is the spontaneous hatching of unfertilized eggs in a normally sexually reproducing species. It has been suggested that tychoparthenogenesis could be the intermediate evolutionary link between sexual reproduction and asexual reproduction via obligate thelytoky (van der Kooi & Schwander, 2015; Schwander, Vuilleumier, Dubman, & Crespi, 2010).

In tychoparthenogenesis, hatching rates and offspring survival are typically much lower than in sexual reproduction (Schwander et al., 2010). A first constraint explaining this low success rate in tychoparthenogenesis is zygote development: restoration of ploidy level, egg activation, and centriole inheritance (Engelstädter, 2008). Developmental problems may vary across lineages and populations, as demonstrated in *Drosophila mercatorum,* where the types of developmental errors were similar but occurred at different proportions among the studied strains (Kramer, Templeton, & Miller, 2002). The second constraint is inbreeding depression: Tychoparthenogenetic offspring have higher levels of inbreeding than sexually produced offspring and may suffer from inbreeding depression due to the genetic load of recessive deleterious mutations which become homozygous (Engelstädter, 2008). The genetic load is expected to be purged by nonrandom mating and genetic drift when deleterious alleles are exposed to selection as homozygotes (Glémin, 2003). Therefore in populations with mating between relatives (or selfing), with small effective size or after bottleneck events, inbreeding depression should be lower and tychoparthenogenesis more successful (Barrett & Charlesworth, 1991; Kirkpatrick & Jarne, 2000). Moreover, in small populations, genetic drift can cause the fixation of deleterious mutations. These fixed deleterious mutations will decrease fitness (i.e., lead to increased genetic load), but they will not contribute to inbreeding depression as there is no allelic variation in the population (Charlesworth & Charlesworth, 1999). Hence in small populations, inbreeding depression is expected to be lower than in large populations both due to purging and due to fixation of mildly deleterious mutations. The magnitude of inbreeding depression could be predicted by the genetic diversity (*H*_E_: expected heterozygosity, Lohr & Haag, 2015), which reflects effective population size, and by the inbreeding coefficient (*F*_IS,_ Glémin, 2003), indicating excess of homozygotes produced by nonrandom mating.

The effect of nonrandom mating to purge the mutation load has been tested in many species via the correlation between inbreeding depression and selfing. In plants, a meta‐analysis found only a weak and nonsignificant negative correlation (Winn et al., 2011). More recently, Dart & Eckert (2013) found higher levels of inbreeding depression in outcrossing populations than in selfing populations of a coastal dune plant. Likewise, in animals, Escobar et al. (2011) found a negative correlation between selfing rates and inbreeding depression in a hermaphroditic mollusks. Evidence for purges of inbreeding depression due to genetic drift has been found in ladybugs in which native populations showed higher inbreeding depression compared to invasive populations that went through a bottleneck in population size (Facon et al., 2011). However, purging by drift is expected to be efficient only for highly lethal recessive alleles but not for mildly deleterious mutations and is less efficient than purging by nonrandom mating (Glémin, 2003; Kirkpatrick & Jarne, 2000).

To study the influence of inbreeding depression in the tychoparthenogenetic performance, we focused on the desert locust, *Schistocerca gregaria*. In this species, as in other locusts, tychoparthenogenesis is automictic thelytoky where ploidy level is restored by endomitosis in the embryo (Goodman, 1978). As a consequence, tychoparthenogenetic embryos may be mosaic for haploid and diploid cells and the majority of embryo cells must be diploid to allow hatching (Pardo, López‐León, Cabrero, & Camacho, 1995; Webb & Komarowski, 1976). This mechanism of ploidy restoration leads to fully homozygous parthenogenetic offspring. Tychoparthenogenesis was first observed in this species by Husain and Mathur (1946) and then further explored by Hamilton (1953, 1955), who obtained four generations of parthenogenetically reproducing locusts under laboratory conditions. He reported an average hatching rate of 25%, compared to around 75% following sexual reproduction. Goodman and Heitler (1977) found a hatching rate of only 10%–30% in laboratory populations of *Schistocerca americana* and *Schistocerca nitens*. More recently, the hatching rates of parthenogenetic eggs were estimated to be 18% in “an established gregarious culture” in the desert locust, *Schistocerca gregaria* (Wang & Sehnal, 2013). Differences in hatching rates among populations were already mentioned in the migratory locust, *Locusta migratoria*, (between a laboratory population and wild populations, Pardo et al., 1995) or in *Drosophila mercatorum* (e.g., between laboratory populations Carson, 1967). Such differences in tychoparthenogenetic capacities between locust populations might be explained by differences in their level of inbreeding depression (faced by fully homozygous parthenogenetic locusts) linked to the history of purging. Indeed, the establishment and maintenance of laboratory populations may involve bottlenecks, small population size and nonrandom mating (e.g., in locusts, Berthier et al., 2010). In Hamilton's study, the similarly high hatching rates of the parthenogenetic offspring in the four successive generations of the desert locust point toward a lack of inbreeding depression in the laboratory population (Hamilton, 1953).

Studying the desert locust, our objectives were (1) to characterize the success of tychoparthenogenetic reproduction in four laboratory populations (among which the one studied by Hamilton, 1953) and (2) to explore the relationship between inbreeding depression and tychoparthenogenetic performance. To this aim, we used four laboratory populations, with varying levels of genetic diversity and of inbreeding coefficient, reared them under parthenogenetic, inbred, and outbred sexual forms of reproduction, and assessed fitness of populations by measuring eight maternal or offspring life‐history traits.

## 2 METHODS

### 2.1 Biological material

We raised four populations of *S. gregaria gregaria* in insect rearing chambers in Montpellier, France, from January to July 2013. These populations originated from the sampling of egg pods in locust laboratories in England, Belgium, France, and Mauritania. All these laboratory lines were first initiated from wild populations of North Africa (Berthier et al., 2010; Pelissié et al., 2016). They correspond to three types of laboratory rearing history: long‐term (England and Belgium), recent (France) and none (Mauritania; Table 1). More precisely, the English population, provided by S. R. Ott (University of Leicester), was inherited from the historic colony of the Anti‐Locust Research Centre in the 1950s and has been submitted to outcrosses with commercial strains since 2009 (BladesBiologicalLtd) (S. R. Ott, personal communication). However, it seems that commercial strains were genetically very similar to the original English laboratory population (see next section). The Belgian population, provided by J. Vanden Broeck (University of Leuven), was based on a few (≤10) founders from a field population (A. De Loof, personal communication). The English and Belgian populations have been reared in the laboratory for >25 years, that is, over 100 generations. The French population was collected from nine egg pods in Mauritania in 2010 and has been bred in the laboratory for seven generations (Pelissié et al., 2016). Finally, Mauritanian population is directly derived from six egg pods collected in the field in February and March 2013 by K. Maeno in Mauritania (JIRCAS, Kyoto University) and was immediately used for the experiment.

**Table 1:** Summary of genetic variability measures of the laboratory populations compared to a reference field population

Population	Year	NG	*N*	*A*_R_	*H*_E_	*F*_IS_
Mauritania (field)	2009	0	21	12.3	0.890	−0.044
France	2011	5	22	8.4	0.840	0.068
Belgium	2009	>100	30	3.9	0.560	−0.005
England	2013	>100	30	3.6	0.5	0.017

Year: year when the studied population was sampled and genotyped; NG: number of generations spent in the laboratory at the date of genotyping; *N*: number of genotyped individuals; *A*_R_, mean allelic richness per locus; *H*_E_: mean expected heterozygosity; *F*_IS_: inbreeding coefficient. Note that except for the English population, genotyping has been performed before this study took place.

### 2.2 Genetic characterization

Based on microsatellite genotyping, we characterized the genetic diversity (expected heterozygosity *H*_E_) and the inbreeding coefficients (*F*_IS_) of the studied populations. The Belgian populations were genotyped in 2009 (Berthier et al., 2010), the French population in 2011 (Chapuis, Plantamp, Streiff, Blondin, & Piou, 2015), and the English population in 2013 for this study. A Mauritanian population sample (different from the population used in this experiment) was genotyped in the past (Chapuis et al., 2014). As the global level of population differentiation was almost null in the wild, this genetic sample can be considered as representative of the experimental population used in 2013. All populations were genotyped with the same six microsatellite loci (DL01, DL06, DL09, DL13, Sgr36, Sgr53: Yassin, Heist, & Ibrahim, 2006; Kaatz, Ferenz, Langer, & Moritz, 2007) using the same ABI 3130 DNA sequencer (Applied Biosystems). Basic population genetic statistics were computed using the R package diveRsity (Keenan, McGinnity, Cross, Crozier, & Prodöhl, 2013). The genotyped wild Mauritanian population had a genetic diversity of 0.89 (Table 1). After five generations in the lab, the French population had a similarly genetic diversity of 0.86 whereas the two old laboratory populations from England and Belgium had lower genetic diversity (0.5 and 0.52, respectively) (Table 1). Inbreeding coefficients were low and nonsignificant for all the genotyped populations. The three laboratory populations were similarly differentiated from one another (from *F*_ST_ = 0.20 between English and Belgian populations to *F*_ST_ = 0.29 between English and French populations), and the French population was the least differentiated from the wild Mauritanian population (*F*_ST_ = 0.08 compared to 0.23 and 0.28 with Belgian and English populations respectively, Annex 1). In addition, as the English population has already been genotyped in 2009, we calculated the genetic differentiation between the two temporal samples, considering that the outcrossing to the commercial line occurred in the intervening time. The low *F*_ST_ (0.02 between the 2009 and 2013 genotypings) suggested that the “outcrossing” had a minimal impact on the genetic diversity of the English laboratory population.

Those results indicated that the French population had a similarly high genetic diversity as the wild Mauritanian population, whereas the English and Belgian populations had substantially reduced genetic diversity, indicating a lower long‐term effective population size and presumably higher genetic drift (Lohr & Haag, 2015). There was no indication of nonrandom mating in any of the studied populations, as revealed by the low and nonsignificant *F*_IS_ (Glémin, 2003). Our expectation was that the two ancient laboratory populations would have lower inbreeding depression (due to genetic drift) and hence higher parthenogenetic capacities compared to the French and the Mauritanian populations.

### 2.3 Parthenogenesis and inbreeding depression experiment

Our goal was to assess, in each population, the effects of inbred reproduction and parthenogenesis, relative to outbreeding, on five offspring traits which may indicate inbreeding depression: the hatching rate of egg masses, the offspring survival 24 hr after hatching, the larval survival until adult molt, the larval development time, and femur length at adulthood. We also measured three maternal traits which indicate the avoidance of inbreeding: the proportion of females laying eggs, the time to first laying, the number of eggs produced by females.

Rearing conditions in the laboratory were 32°C and 50% humidity with a photoperiod of 13/11 hours (Pelissié et al., 2016). The first experimental generation is composed by full‐sib families that were made of the egg pods collected from each population (*n* = 8; 8; 7 and 6 for England, Belgium, France, and Mauritania, respectively). Siblings from those families were raised as a group in plexiglass cages; at 6 weeks (i.e., ~2.5 weeks after adult molt), males and females were separated, to prevent any uncontrolled mating. At 9 weeks, when sexual maturity was supposed to be achieved for all populations (Uvarov, 1966), three to 12 females per family were isolated and three reproduction treatments were applied: a “parthenogenetic” treatment where females were not offered a mate, an “inbreeding” treatment where brothers were offered as mates, and an “outbreeding” treatment where a male from a different family within the same population was offered as a mate. Females and their mates were placed in the same 1‐L box for 24 hr, and then separated for 48 hr to allow females to lay eggs. Females had access to a laying tank containing wet, sterilized sand, where they could bury egg pods. If after that period the female had not yet laid eggs, she was mated with the same male for another 24 hr. This process was repeated until the mating was successful. The time elapsed from the beginning of the experiment (9‐week‐old females) to first egg laying was recorded, although with a limit of 1 month due to time constraints. Only two pods per females (if produced) were kept. Hatchlings were fed as soon as they emerged (~12 days after laying). Twenty‐four hours after emergence, we counted the number of living individuals, the number that had emerged from their eggs but immediately died, and the number of eggs that did not hatch to calculate for each pod the number of eggs, the hatching rate and the survival 24 hr after hatching. As the only Mauritanian female in the parthenogenetic treatment which laid egg pods did so on top of the sand and the egg pods dried to the point of inviability, it was not possible to assess the number of eggs, the hatching rate and the offspring survival 24 hr after hatching in this population.

Twelve to 15 offspring per egg pods (i.e., 698 offspring) were then reared in individual 1‐L boxes from hatching to adult molt to assess two more life‐history traits. For practical timing constraints, as the Mauritanian population was obtained late in the experiment, this measure was carried out in the three other populations only (England, Belgium and France). From the first egg pod, 12 individuals were isolated into separate 1‐L boxes with a pierced lid and observed until they reached adult molt. They were monitored daily to record larval survival and larval development time (until adult molt). When offspring reached adulthood, we also measured the femur length, which is a good proxy of the body size given its low measurement error (e.g., Chapuis et al., 2017). Between the third and fourth instars, desert locusts can undergo an extramolt, which leads to six rather than five instars. This extramolt is a catch‐up growth strategy allowing small‐size offspring to increase their body size at adult molt, at the cost of a longer development time (Pelissié et al., 2016). Therefore, for each individual we recorded the number of larval molts and used it as an explanatory variable. Details on the number of individuals (female adults and offspring) measured in each population and each treatment are given in Annex 2.

### 2.4 Statistical analyses

All statistical analyses were performed on R.3.2.3 (R Core Team 2015). We tested the interaction effects of “population” and “reproduction treatment” on the proportion of females laying eggs and on the time to first laying using a generalized linear model with a binomial family and a quasi‐poisson family (to account for the overdispersion), respectively. Similarly, we tested the effects of “population,” “reproduction treatment” and “egg pod rank” (first or second), and every simple interaction between pairs of factors, on the number of eggs produced per pod, using a linear model with Gaussian distribution, and on the hatching rate and the survival 24 hr after hatching, using a generalized linear model with quasi‐binomial distribution (Zuur, Ieno, Walker, Saveliev, & Smith, 2009). Finally, larval development time and adult femur length were analyzed using generalized linear models with quasi‐poisson distribution and a linear model. In each model, we tested the effects of “population,” “reproduction treatment,” “sex” and “extra molting” and every simple interaction between pairs of factors. For each analysis, we performed a backward stepwise model selection by AIC and tested the significance of factors present in the selected model using a chi‐square tests for binomial distributed variables or a *F* test for Gaussian, quasi‐poisson, and quasi‐binomial distributed variables. Significant factors were explored using Tukey HSD post hoc tests. Finally, survival (in days) from the 2nd day after hatching to adult molt was modeled using a Cox proportional hazards model (Therneau, 2012; Therneau & Grambsch, 2000). “Reproduction treatment” and “population” were fitted as fixed effects. All analyses were also run using mixed effect models with family as random effect (to account for the nonindependence of the data) but as it yielded the same results, we decided to present only the generalized linear models.

## 3 RESULTS

### 3.1 Mother traits

**Figure 1:**
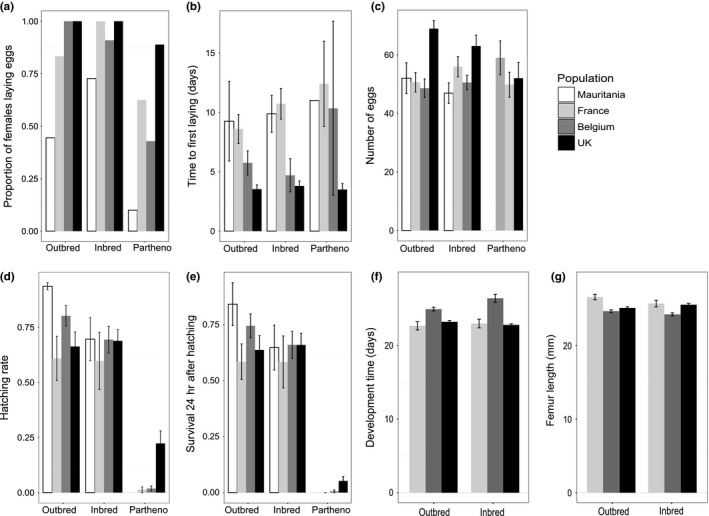
Means and standard deviations of measured traits in populations and reproductive treatments. (a) Proportion of females laying eggs, (b) time to first egg laying, (c) number of eggs, (d) hatching rate in one egg pod, (e) offspring survival 24 hr after hatching, (f) larval development time, and (g) adult femur size. Measurements (a) to (e) were made on the four studied populations (Mauritania, France, Belgium, and England) whereas measurements related (f) and (g) could be measured from Belgium, France, and England for the outbred and inbred treatments only. The genetic characteristics and the number of individuals measured in each population are detailed in Table 1, Annexes 1 and 2

The proportion of females laying eggs was not different between outbred and inbred treatment, except for the French population (higher proportion in the inbred treatment: χ²_1_ = 2.778 and *p* = .027), and was significantly lower in parthenogenetic treatments in all populations (Table 2, Figure 1a). The time to first laying and the number of eggs produced were not affected by the reproduction treatment (Table 2, Figure 1b,c).

**Table 2:** Factors significantly influencing maternal and offspring traits

Trait	Selected parameters	*df*	*F* value deviance	*p*‐value
Laying probability	Population	3	30.68	.000
	Treatment	2	23.52	.000
Time to first laying	Population	3	124.32	.000
Egg number	Population	3	9.92	.000
	Treatment	2	2.18	.116
	Egg pod	1	11.99	.001
	Population:treatment	5	2.03	.077
Hatching rate	Population	3	1.23	.299
	Treatment	2	45.04	.000
	Population:treatment	5	2.61	.027
First day survival	Treatment	2	60.56	.000
Development time	Population	2	72.3112	.000
	Sex	1	34.9112	.000
	Extramolting	1	37.9551	.000
	Population:treatment	2	6.694	.001
Femur length	Sex	1	1152.22	.000
	Population	2	77.58	.000
	Extramolting	1	11.42	.001
	Population:treatment	2	4.39	.013

For each trait, we reported the degrees of freedom, *F* values or deviance (for chi‐square tests) and *p*‐values. Note that for development time and femur length only three populations (Belgium, France and UK) and two treatments (outbreeding and inbreeding) were analyzed, see section 2 for details.

Population had an effect on proportion of females laying eggs, time to first laying, and egg number (Table 2, Figure 1a–c). These population effects remained even when the parthenogenetic treatment was omitted. For instance, Mauritanian females were less prone to lay eggs whereas English females had shortest time from mating to first laying and laid significantly more eggs. In the parthenogenetic treatment, the English population had the highest proportion of females laying eggs. Moreover, egg pod rank had a significant effect in the variation of egg pod size: The first egg pod had significantly more eggs than the second (as already reported in gregarious lines: (Maeno & Tanaka, 2008).

### 3.2 Offspring traits

Hatching rate and survival 24 hr after hatching were not different between inbred and outbred treatments (Table 2, Figure 1d,e). Those two traits were severely decreased in the parthenogenetic treatment. There was no effect of population on hatching rate and first day survival. However, in the parthenogenetic treatment, the English population had the highest hatching rate and first day survival.

**Figure 2:**
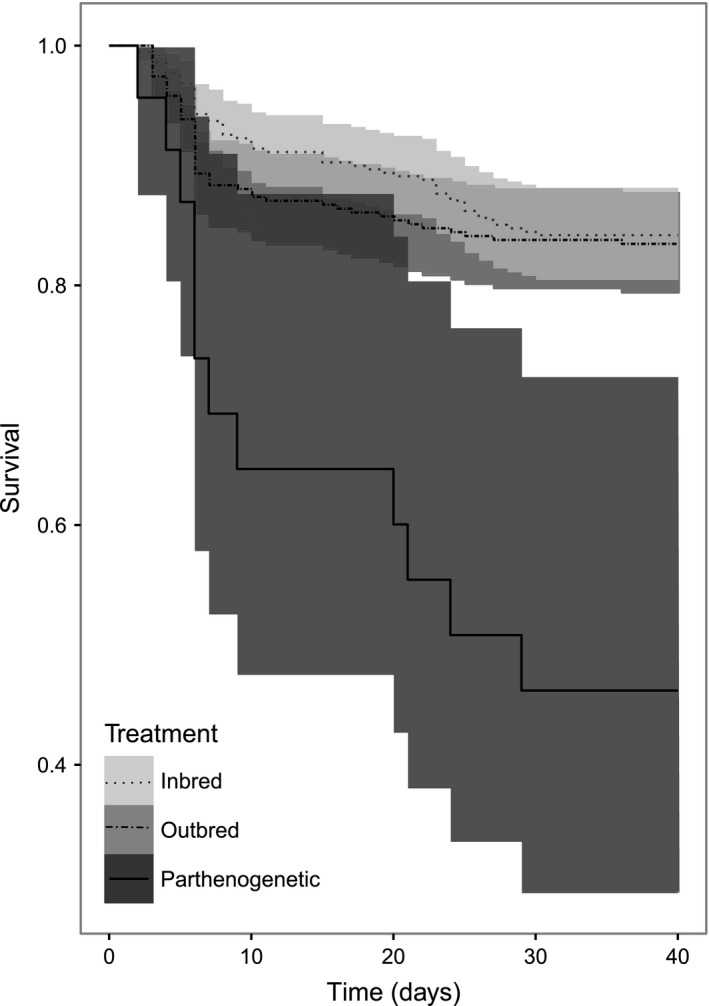
Larval survival curves of inbred, outbred, and parthenogenetic offspring. Inbred and outbred offspring are from the French, Belgian, and England population whereas the parthenogenetic offspring are from the England population only. Central survival curves are surrounded by 95% confidence intervals

In English, Belgian, and French populations (Mauritanian offspring were not reared until adult molt), we found no difference in survival at the larval stage between the inbred and outbred treatments (Figure 2). Moreover, there were significant interactions between population and treatment in larval development time and femur length (Table 2). More precisely, inbreeding significantly increased larval development time only in Belgian offspring (Figure 1f; *F*_1,204_ = 7.59; *p* = .006). Similarly, inbreeding had a significant positive effect on femur length in English adult offspring (*F*_1,281_ = 12.91; *p* = .0004) but a negative effect in French and Belgian adult offspring (Figure 1g; *F*_1,42_ = 7.58 and *p* = .009; *F*_1,199_ = 6.63; *p* = .011, respectively).

Note that, although we initially reared 698 offspring, only ten survived to adulthood in the parthenogenetic treatment, all from the English population (Annex 2). In this population, being born to a parthenogenetic mother significantly raised mortality risk (Figure 2, χ²_2_ = 15.40; *p* = .0005). The 10 surviving parthenogenetic offspring had significantly longer larval development time and reached larger adult body sizes than the offspring in the two other treatments (*F*_2,294_ = 9.19; *p* = .0001 and *F*_2,289_ = 18.19; *p* < .0001).

Population of origin had a significant effect on development time and size, with the Belgian population taking longer to reach the each molt stage and having smaller size at adulthood than the French or English populations in both outbred and inbred treatments (Table 2 and Figure 1f,g). Finally, as expected, sex and extramolting had a significant effect on larval development time and on adult femur length, such that extramolting females had the longest development time and biggest size (Table 2; Pélissié et al., 2016).

## 4 DISCUSSION

### 4.1 Inbreeding depression

Contrary to our expectations, the various demographic histories of the four studied populations did not translate into a clear pattern of differences in inbreeding depression. Among the five measured offspring traits, only two showed signs of inbreeding depression, which were not consistent with our predictions of a lower inbreeding depression and/or a lower level of genetic diversity. Indeed, our experiment revealed signs of inbreeding depression in larval development time and adult femur length in the Belgian population and in adult femur length in the French population. On the contrary, the Mauritanian and English populations showed no sign of inbreeding depression and inbreeding even had a positive effect on adult femur length in the English population. The lack of inbreeding depression may either be due to purging of the genetic load and fixation of mildly deleterious alleles in some populations or to experimental flaws.

First, in the English population, which does not show signs of inbreeding depression, the level of genetic diversity was the lowest, indicating smaller long‐term effective population size, such that potential genetic drift may have purged the genetic load or fixed deleterious alleles. However, this does not seem to apply for the Belgian population, which showed inbreeding depression but otherwise had similar genetic diversity as the English population. In the Mauritanian and French populations, with similarly high genetic diversity, the former showed no inbreeding depression whereas inbreeding depression is observed in terms of adult body size in the later. Alternatively, inbreeding depression may pass undetected for several reasons. First, statistical analyses may lack power, either because of the limited sample size or the limited variation of the inbreeding coefficient between offspring produced by outbred or inbred crosses. For example, the lower genetic diversity in the English and Belgian populations may have severely reduce differences in inbreeding coefficients between offspring from outbred and the inbred treatments. Second, a limited number of traits were explored, while traits may be differentially affected by inbreeding depression (Hedrick & Kalinowski, 2000). We selected traits that were more or less closely related to fitness, but it would have been ideal to have a proper measure of fitness (e.g., Facon et al., 2011). Furthermore, traits appearing earlier or later in life are predicted to display contrasted responses to inbreeding. Theoretical models indicate that highly recessive deleterious mutations, when made homozygous by inbreeding, are expected to cause marked declines in fitness very early in the life cycle and are therefore readily purged by inbreeding (or by tychoparthenogenesis). In contrast, mildly deleterious mutations may often cause inbreeding depression later in the life cycle and remain recalcitrant to purging (Dart & Eckert, 2013). Accordingly, in our experiment, slight but significant inbreeding depression was detected for later‐life traits in the French and Belgium populations (i.e., larval development time and adult femur length) while early‐life traits like hatching rate were unaffected. Additionally, the three mother traits (i.e., egg‐laying proportion, time from mating to first laying, egg number) did not show any sign inbreeding avoidance, and we even found a positive effect of inbred mating on the proportion of females laying eggs in the French population. Theory predicts that under strong inbreeding depression, individuals should be more reluctant to invest in inbred mating (Tsitrone, Duperron, & David, 2003). Therefore, the low inbreeding depression could have resulted in an absence of inbreeding avoidance.

### 4.2 Success of tychoparthenogenesis

In the four studied populations, the parthenogenetic offspring have lower hatching rate, survival 24 hr after hatching, larval survival and development time than offspring from the other treatments. As expected, we found drastic differences in the tychoparthenogenetic capacities between the laboratory populations of *S. gregaria*. The English population, which derived from the population originally studied by Hamilton 60 years earlier (Hamilton, 1953), was the most successful in producing parthenogenetic offspring. In addition, similarly to Hamilton's results, the females formed by parthenogenesis in the English population were able to produce a second generation of parthenogenetic eggs (data not shown). In comparison, in the three other populations a lower percentage of females produced parthenogenetic eggs and no hatchling survived until adult molt, or even hatched in the case of the Mauritanian population.

The lower fitness of the parthenogenetic offspring was expected under our hypothesis as they are fully homozygous and should face larger inbreeding depression than the inbred offspring. Following this line of reasoning, it is possible to roughly estimate number of lethal alleles in the haploid genome (number of lethal equivalents; Archetti, 2004). Assuming a Poisson distributed number of lethal alleles, the probability of hatching for a parthenogenetic offspring is the probability of having zero lethal allele in the haploid genome. Using the hatching rate of parthenogenetic offspring relative to outbred offspring, we estimated 1.1, 3.8, and 3.9 lethal alleles in the haploid genome (in the English, Belgian, and French populations, respectively). Those estimates are in the range of previously published results but are larger than those published in insects (between 0.48 and 0.77 lethals equivalents in three *Drosophila* species, Lynch & Walsh, 1998). Using those estimates of number of lethal equivalent, we calculated the potential hatching rates of inbred offspring relative to outbred offspring. We obtained 38%, 39%, and 76% relative hatching rates which were lower than the measured values (98%, 86%, and 100% in the English, Belgian, and French populations, respectively). We conclude that inbreeding depression itself is not sufficient to explain the very low fitness of the parthenogenetic offspring and the differences in parthenogenetic performance among all four studied populations.

The alternative hypothesis explaining this low success rate in tychoparthenogenesis is that the lack of inbreeding depression is probably only one necessary step toward efficient tychoparthenogenesis. The other steps may be related to the developmental constraints. As mentioned before, diploidy restoration proceeds by endomitosis in the embryo which may be mosaic for haploid and diploid cells and, it seems that the majority of embryo cells must be diploid to allow hatching (Pardo et al., 1995; Webb & Komarowski, 1976). Under this hypothesis, it seems that the English population had overcome such constraints during its long rearing history. The greater parthenogenetic capacities of the English population may be caused by a specific laboratory adaptation or to genetic drift in the successive generations. Accordingly, there is a fairly high level of genetic differentiation at microsatellite markers (*F*_ST_) as well as phenotypic divergence between all populations. For example, the English population had shorter time to first laying (in all reproduction treatments) and produced more eggs than the other populations. Further work would be needed to confirm that the developmental errors differ between the studied populations.

### 4.3 Implications for locust population dynamics

In 1953, based on the relatively high success of tychoparthenogenetic reproduction measured in his laboratory population of the desert locust, Hamilton stated that “thelytokous parthenogenesis may play a part in the preservation of the species when isolated in the solitary phase” (Hamilton, 1953). Indeed, during recession periods, solitarious females may fail to find a mate among sparsely distributed males, and tychoparthenogenesis could be adaptive (Uvarov, 1966). Based on a theoretical model, Schwander et al. (Schwander et al., 2010) even proposed that when populations were at low density and females were mate‐limited, a feedback loop could lead to a fast reduction in the ratio of males in the population and to an increased selection for tychoparthenogenetic.

However, we showed that, in the desert locust, several traits are negatively impacted by parthenogenesis, thus limiting the potential adaptive advantage of this reproductive strategy. Furthermore, in agreement with previous studies, our laboratory results indicated that viable offspring were not successfully produced by tychoparthenogenesis, with the single exception of the same long‐term laboratory population already known for this outcome (Hamilton, 1953). This suggests that rates of tychoparthenogenesis may be very low in nature. This is corroborated by indirect molecular approaches that did not reveal any tychoparthenogenetic offspring (i.e., fully homozygous, see Booth et al., 2012) within large numbers of wild desert locusts genotyped so far (Chapuis et al., 2014, 2017). Altogether, our results, with their methodological restrictions, did not bring evidence for an important adaptive role of tychoparthenogenesis in newly solitarized populations of the locust.

## CONFLICT OF INTEREST

None declared.

## AUTHOR CONTRIBUTIONS

CJL, LB, EC, and HJ‐P ran the experiments and the analyses. LB and MPC performed the microsatellite genotyping and analyses. CJL, EC, HJ‐P, and MPC wrote the manuscript. EC and HJ‐P provided equal contribution to this work.
