# Supplementary material for: Exploring the relationship between tychoparthenogenesis and inbreeding depression in the Desert Locust, Schistocerca gregaria
Source: Ecol Evol. 2017 Jun 28;7(15):6003–11. doi: 10.1002/ece3.3103 (PMC5551105; doi:10.1002/ece3.3103)
Supplement: Supplementary file 1 [file ECE3-7-6003-s001.doc]

**Annex 1:** Genetic differentiation (F_ST_) between the studied populations

| Population | Belgium | England | France |
| --- | --- | --- | --- |
| Mauritania (field) | 0.230 | 0.287 | 0.085 |
| France | 0.239 | 0.292 |  |
| England | 0.206 |  |  |

**Annex 2:** Sample size (number of female adults and number of offspring reared until adult molt) within each population and each treatment.

| Population | Treatment | Nb of females | Nb of offspring |
| --- | --- | --- | --- |
| Belgium | inbred | 11 | 90 |
|  | outbred | 12 | 120 |
|  | parthenogenetic | 7 | 0 |
| France | inbred | 11 | 21 |
|  | outbred | 12 | 26 |
|  | parthenogenetic | 8 | 0 |
| Mauritania | inbred | 11 | 0 |
|  | outbred | 9 | 0 |
|  | parthenogenetic | 10 | 0 |
| England | inbred | 15 | 144 |
|  | outbred | 15 | 145 |
|  | parthenogenetic | 9 | 10 |
| Total |  | 130 | 556 |
